# Endothelial Dysfunction Markers Correlate with the Time Since Completion of Tuberculosis Treatment and the Number of Previous Tuberculosis Episodes

**DOI:** 10.3390/idr17020021

**Published:** 2025-02-28

**Authors:** Chrisstoffel Jumaar, Steve Jacobs, Carmen Payne, Olakunle Sanni, Elize Louw, Nicola Baines, David Maree, Benjamin Botha, Merga Belina Feyasa, Hans Strijdom, Brian Allwood, Gerald J. Maarman

**Affiliations:** 1 Centre for Cardio-Metabolic Research in Africa, Division of Medical Physiology, Department of Biomedical Sciences, Faculty of Medicine & Health Sciences, Stellenbosch University, Cape Town 8000, South Africa; 27421309@sun.ac.za (C.J.); stevejacobs@sun.ac.za (S.J.); kunlesola2810@gmail.com (O.S.); jgstr@sun.ac.za (H.S.); 2 Division of Pulmonology, Department of Medicine, Faculty of Medicine & Health Sciences, Stellenbosch University and Tygerberg Hospital, Cape Town 8000, South Africa; ehlouw@sun.ac.za (E.L.); nicolabaines@sun.ac.za (N.B.); brianallwood@sun.ac.za (B.A.); 3 Cape Winelands TB Centre, Brewelskloof Hospital, Worcester 6849, South Africa; benjamin.botha@westerncape.gov.za; 4 Division of Epidemiology and Biostatistics, Department of Global Health, Stellenbosch University, Cape Town 7530, South Africa; merga.belina@aau.edu.et; 5 CDT—Africa Center of Excellence, College of Health Sciences, Addis Ababa University, Addis Ababa 10001, Ethiopia

**Keywords:** tuberculosis, endothelial dysfunction, vascular remodeling, post-tuberculosis lung disease, PTLD, pulmonary hypertension

## Abstract

**Background:** Despite “successful” treatment, some lung tuberculosis (TB) patients develop long-term lung impairments that includes damage to the parenchyma and reduced function, which may predispose them to diseases like pulmonary hypertension. However, this is not well understood. Therefore, we investigated whether previous or current TB patients would display elevated biomarkers of endothelial dysfunction and vascular remodeling. **Methods**: We performed assays for ADMA, VCAM-1, VEGF, angiopoietin-1, TBARS, NT-pro-BNP, and cardiac troponin-I. We further stratified the patients based on 1, 2, 3, and >3 previous TB episodes, and 1–5 yrs, 5–10 yrs, 10–15 yrs and >15 yrs after the last TB treatment completion. We also assessed correlations between the biomarkers and the number of previous TB episodes or the time since the completion of the last TB treatment. **Results**: ADMA was 70 times higher, VEGF was 2000 times higher and angiopoietin-1 was 6500 times higher than the normal range. NT-pro-BNP and cardiac troponin-I were undetected, and TBARS levels were low. There was a positive linear relationship between the number of previous TB episodes and angiopoietin-1, and between VEGF and the number of previous TB episodes. ADMA, VCAM-1 and TBARS exhibited a weak and negative linear association with the number of previous TB episodes. A negligible negative linear association was observed between the time since the completion of the last TB treatment and angiopoietin-1, VEGF and ADMA. **Conclusions**: Therefore, having >1 previous TB episode, despite the successful completion of TB treatment, associates with an increased risk of endothelial dysfunction/angiogenesis or vascular remodeling.

## 1. Introduction

It is estimated that 25% of the global population is infected with lung tuberculosis (TB), with the majority remaining asymptomatic [1,2]. The World Health Organization (WHO) estimates that there was an increase of 4.5% in people who contracted TB from 10.0 million in 2020 to 10.6 million in 2021. This number increased to 10.8 million (95% uncertainty interval [UI]) in 2023, an increase from 10.7 million in 2022 [2]. The incidence rate also increased by 3.6%. The latest WHO global report (2024) indicates that the TB incidence rate was 134 per 100,000 population, a 0.2% increase when compared with 2022 [2]. The incidence rate also increased by 3.6% in the same period. It was estimated that the incidence of TB ranges from less than 10 cases per 100,000 population in developed nations to more than 500 in high TB-burden countries, including South Africa [3]. In South Africa, there was an increase in the incidence to 615 per 100,000 population in 2019 as compared to the WHO 2015 data of 520 per 100,000 population [4,5].

In 2020–2024, the WHO estimated an 85% global treatment success rate for newly diagnosed TB cases and a 76% success rate for those living with HIV. The overall global success rate was 80.1%, and 78.9% on the African continent. However, despite successful treatment, some patients develop serious long-term lung complications. These include parenchymal (tuberculoma, cavitation and aspergilloma), airway disease (bronchiectasis, tracheobronchial stenosis, chronic obstructive pulmonary disease (COPD) and broncholithiasis), as well as diseases of the pleural or chest wall (empyema, fibrothorax, pneumothorax and bronchopleural fistula), mediastinum (calcified lymph nodes, fibrosing mediastinitis and pericardial TB) and vascular system (Rasmussen aneurysm, arteritis and thrombosis and dilated bronchial arteries) [6,7,8]. It is not well understood why patients develop these complications, but there is a need for studies that characterize the involvement of molecular pathways, such as endothelial dysfunction/angiogenesis, that may drive the pathology, especially given the vascular complications post-TB. Therefore, the present study investigates the presence of endothelial dysfunction/angiogenic markers in a post-TB clinical context.

## 2. Materials and Methods

This study was nested within a larger and ongoing clinical study, the PuPPeT II trial, which is a cross-sectional study that evaluates the prevalence of pulmonary hypertension across different TB populations [9]. The parent study consisted of four different TB populations, whereas the present study is a sub-analysis with a fraction of the population. The inclusion criteria for the parent study required that participants be 18 years of age or older and that they provide written consent to participate. Exclusion criteria for the study were failure to provide written and informed consent, multidrug-resistant or extensively drug-resistant TB patients, patients with active malignancy, the presence of dementia or delirium, if the patient was deemed medically unstable by the study team, or if any condition was present that may have prevented clinical investigations. For the present study, patients with a history of TB (1–5 previous TB episodes and up to 15 years post-TB after successful TB therapy) were recruited. Ethical approval was obtained from Stellenbosch University’s Human Research Ethics Committee (N18/08/091).

### 2.1. Phlebotomy and Serum Collection

After informed consent was obtained, blood was collected in vacutainer blood tubes (VACUCARETM, Midrand, South Africa). Plasma blood collection tubes contained ethylenediaminetetraacetic acid, which blocks the coagulation cascade by binding to calcium ions, while serum collection tubes were used for the isolation of serum samples. The serum collection tubes (VacuLab^®^ SSGT tubes, Jiangsu, China) were coated with a clot activator and gel, which allows for serum separation and thus high-quality serum specimens for laboratory analysis. Specimens were taken to the laboratory, where all vacutainers of blood were centrifuged at 3000 rpm for 10 min. The centrifuge (Merck, Eppendorf^®^ Centrifuge 5810, Cape Town, South Africa) was solely used for TB work by the Division and all procedures were performed in a Biosafety Cabinet (BSC) Class II (Centrifugal fan H14 HEPA filter) (BIOBASE, Shandong, China). Plasma and serum were stored as 50 microlitres (μL) aliquots at −80 °C in a freezer designated for potentially biohazardous specimens. All blood was handled inside the BSC according to the Center for Disease Control and Prevention guidelines.

### 2.2. Asymmetrical Dimethylarginine (ADMA)

The reagents were first brought to room temperature (18–25 °C). The wash buffer solution was prepared by diluting 30 mL of the concentrated buffer with 720 mL of distilled water to provide a working solution of 750 mL. The standard was made up by centrifuging the standard at 10,000× *g* for one minute and adding 1 mL of the reference standard and sample diluent to prepare a 1000 ng/mL working solution. After allowing the solution to stand for 10 min, it was meticulously inverted until fully dissolved. Serial dilutions were prepared as recommended in the manufacturer’s protocol. Hereafter, the biotinylated detection antibody solution, diluent and horseradish peroxidase (HRP) conjugate solution were added as per guidelines. The plate was sealed and incubated at 37 °C for 30 min. The FLUOstar^®^ Omega Microplate reader (FDA 21 CFR Part 11, BMG Labtech, Ortenberg, Germany) was used to measure the optical density at 450 nanometres (nm) [10].

### 2.3. Vascular Cell Adhesion Molecule-1 (VCAM-1)

The reagent kit was prepared according to the manufacturer’s instructions. All the reagents were brought to room temperature (18–25 °C) after which the wash buffer, standard working solution, biotinylated detection antibody and HRP conjugate working solution were prepared. We started by adding 100 μL of the different concentrations of the diluted standard, blank and patient samples to the wells. The plate was sealed and incubated for 90 min at 37 °C. The liquid was decanted followed by the immediate addition of 100 μL of the Biotinylated Detection Ab working solution. The plate was covered with a new sealer and incubated for 60 min at 37 °C. Post incubation and decantation of the liquid, 350 μL of wash buffer was added to each well, allowing it to soak for one minute, after which the liquid was decanted, and the plate was dried by careful patting against clean absorbent paper. This step was repeated for three cycles. We proceeded to add 100 μL of HRP Conjugate working solution to each well, sealed the plate and incubated for 30 min. After incubation, the liquid was decanted and the previously mentioned wash step was repeated five times. We added 90 μL of Substrate Reagent to the wells, covered the plate with a new sealer and proceeded to incubate it for another 15 min, taking care to protect the plate from any light exposure. At this stage, the FLUOstar^®^ Omega Microplate reader was also preheated for analysis. After the final incubation step, 50 μL of the stop solution was added to the wells and the optical density was determined with a microplate reader at 450 nm [11].

### 2.4. Procartaplex Kit

The kit was reconstituted according to the manufacturer’s guidelines to measure markers like VEGF, angiopoietin-1, NT-pro-BNP and cardiac troponin-I. The Luminex kit and all components were brought to room temperature. The Standards, Wash buffer, Detection Ab and magnetic beads were prepared. To the wells, 25 μL of the universal assay buffer was added, followed by 25 μL of sample or standards, while background wells only contained 50 μL of the 1× universal assay buffer. The plate was sealed and incubated with a shaking feature activated for 60–120 min at room temperature. Hereafter, 25 μL of the detection antibody was added to the wells, the plate was resealed and incubated (with shaking action) for 30 min at room temperature. The plate was washed twice in an automated plate washer (Biotek, 50TS software, Agilent, Santa Clara, CA, USA), with the wash buffer, followed by the addition of 120 μL reading buffer and a further incubation period of five minutes at room temperature. Thereafter, the plate was analyzed using the Magpix Luminex instrument (Bioplex Manager 6.1, Thermofisher, Cape Town, South Africa). The final data were expressed as ng/mL.

### 2.5. Thiobarbituric Acid Reactive Substances Assay

The thiobarbituric acid reactive substances (TBARS) assay is a measure of lipid peroxidation. It measures malondialdehyde, a reactive compound formed during lipid peroxidation, caused by ROS. The method by Jentzsch et al. (1996) was used [12]. First, 50 μL of blood serum was added to an Eppendorf tube and mixed with 6.25 μL butylated hydroxytoluene (4 mM, in ethanol) and 50 μL of ortho-phosphoric acid (0.2 M). Thereafter, the tubes were whirled for ten seconds, followed by the addition of 6.25 μL of thiobarbituric acid reagent. The samples were whirled for another ten seconds before being micro-centrifuged for two minutes at 3000 rpm (4 °C) to allow the volumes to collect at the bottom of the Eppendorf tubes. The samples were then placed onto a heating block for 45 min, pre-heated to 90 °C. Thereafter, the samples were placed on ice for two minutes followed by five minutes at room temperature. Subsequently, 500 μL of n-butanol was added to each sample, followed by the addition of 50 μL of saturated sodium chloride to each sample and whirled once again for ten seconds. The samples were then micro-centrifuged at 12,000 rpm for two minutes at 4 °C, allowing the separation of the samples into a transparent top butanol phase and a white bottom phase containing the protein pellet. Of this top-butanol phase, 300 μL was pipetted into each well of a UV-readable Greiner 96-well plate (Lasec, Cape Town, South Africa). Samples were read at an absorbance of 532 nm on the FLUOStar^®^ Omega plate reader (Version, BMG LABTECH, Ortenberg, Germany) with Omega software (FDA 21 CFR Part 11). The final concentration of malondialdehyde in the samples was calculated using the Beer–Lamberts law and the extinction coefficient of 1.54 × 10^5^ M^−1^/cm^−1^. Final values were expressed as μmol malondialdehyde/mL serum [13].

### 2.6. Statistical Analyses

Statistical analyses were performed using GraphPad Prism 9. The data underwent normality testing using the Shapiro–Wilk test. If the data had a normal distribution, unpaired *t*-tests were performed. If data were not normally distributed, we opted for a non-parametric inference of comparing the medians using a quantile regression model. Statistical significance was considered as *p* < 0.05. 

## 3. Results

### 3.1. Descriptive Data of the Patients

The mean age of the patients was 45 years; 4 (10.0%) were 18–29 years, 10 (25.0%) were 30–39 years, 9 (22.5%) were 40–49 years, 12 (30.0%) were 50–59 years and 5 (12.5%) were ≥60 years (Table 1). In terms of sex, 15 (37.5%) were female, and 25 (62.5%) were male. In terms of smoking status, 21 (52.5%) were current smokers at the time of enrolment, 13 (32.5%) were ex-smokers, and 6 (15.0%) had never smoked. Only 5 (12.5%) were HIV positive, 11 (27.5%) had systemic hypertension, and 6 (15.0%) had heart disease (Table 1).

### 3.2. Distribution of Biomarkers

Descriptive results are presented in Table 2. Angiopoietin-1 ranges from a minimum of 236.6 to a maximum of 3226.3 with a mean of 1391.098. VEGF ranges from a minimum of 68.7 to a maximum of 2.193.9 with a mean of 562.108. ADMA ranges from a minimum of 547.7 to a maximum of 883.1 with a mean of 693.638. VCAM-1 ranges from a minimum of 25.2 to a maximum of 83.5 with a mean of 62.275. TBARS ranges from a minimum of 0.31 to a maximum of 0.61 with a mean of 0.448.

### 3.3. Correlations Data

Table 3 illustrates the positive linear correlation between some biomarkers and the number of previous TB episodes. It shows a notable positive linear relationship between the number of previous TB episodes and angiopoietin-1, as well as between VEGF and the number of previous TB episodes. Conversely, the remaining biomarkers (ADMA, VCAM-1 and TBARS) exhibit a weak and negative linear association with the number of previous TB episodes. Table 4 demonstrates a negative linear correlation between certain biomarkers and the time elapsed since the last TB treatment. There is a negligible negative linear association observed between the time since the completion of the last TB treatment and angiopoietin-1, VEGF and ADMA. In contrast, VCAM-1 and TBARS, among the remaining biomarkers, display a weak yet positive linear relationship with the time since the completion of the last TB treatment.

## 4. Discussion

The main findings of this study were that, compared with their respective normal values, ADMA was approximately 70 times higher, VEGF 2000 higher and angiopoietin-1 was 6500 times higher. The NT-pro-BNP and cardiac troponin-I were undetected, and TBARS levels were low. There was a positive linear relationship between the number of previous TB episodes and angiopoietin-2, as well as between VEGF and the number of previous TB episodes. Conversely, the remaining biomarkers (ADMA, VCAM-1 and TBARS) exhibited a weak and negative linear association with the number of previous TB episodes. There was a negligible negative linear association observed between the time since the completion of the last TB treatment and angiopoietin-1, VEGF and ADMA. In contrast, VCAM-1 and TBARS, among the remaining biomarkers, displayed a weak yet positive linear relationship with the time since the completion of the last TB treatment.

ADMA is an analogue of L-arginine and a metabolic product which occurs naturally in circulation. It is considered to be the natural inhibitor of nitric oxide (NO) synthase, and when present in high concentrations, it results in a decrease in NO production. NO is an important vasodilator as well as an inhibitor of inflammation in the vasculature by suppressing the actions of adhesion molecules and inflammatory mediators [14]. Whereas, high ADMA levels are often an indication of abnormal endothelial function [12,14]. Moreover, ADMA is a risk marker of cardiovascular disease, and high ADMA levels are observed in patients with hypertension, type-2 diabetes and atherosclerosis [14]. Our data demonstrated that ADMA levels were similar between the two populations. According to our knowledge, there are no studies that have measured ADMA in patients with active TB; therefore, we cannot make that comparison with our data. However, previous studies showed that ADMA in healthy individuals are ~10 ng/mL [15], meaning that the ADMA in our populations was approximately 70 times higher (697 ng/mL) than the normal range. We did not measure NO in our study, due to the volatile nature of the molecule, but our ADMA findings suggest that patients who are post-TB may have a reduced vasodilatory capacity. This is known to predispose to diseases such as pulmonary hypertension, meaning that TB patients who were previously treated for TB and have had at least one TB episode previously may be at risk of pulmonary vascular abnormalities, despite treatment.

VCAM-1 is an endothelial cell adhesion molecule, identified as a regulator of cellular adhesion in inflammation and a mediator of migration of leukocytes, such as macrophages and T-lymphocytes, across the endothelium [16,17]. VCAM-1 is a marker of endothelial dysfunction and is upregulated in murine TB and pulmonary hypertension [18,19,20]. Furthermore, the expression of VCAM-1 is upregulated in the presence of pro-inflammatory cytokines, such as tumor necrosis factor-alpha, reactive oxygen species and oxidative stress [17,21]. Our patients had low levels of oxidative stress. TBARS are produced as by-products during lipid peroxidation, and the TBARS assay particularly measures the concentration of malondialdehyde [22]. Our data effectively suggests that oxidative stress in the form of lipid peroxidation is not a role player in the post-TB context in our study population. This contrasts with previous studies that have shown elevated lipid peroxidation in TB patients. However, this contradicting finding could be explained by the fact that the current study’s patients were all post-TB and post-treatment, suggesting that the lipid peroxidation might have cleared. Moreover, one should keep in mind that perhaps better markers of oxidative damage could have been assessed, including F2 isoprostanes, advanced oxidation protein products and 8-hydroxy-2′-deoxyguanosine [23], which have been previously linked with the post-TB disease state.

VEGF is an important factor which plays a role in the promotion of angiogenesis. Its function in these processes encompasses cellular proliferation, anti-apoptotic activities, increasing the permeability of the blood vessels, vasodilatation as well as signaling of inflammatory cells [24]. The mean concentration of VEGF in our study was approximately 2000 higher than the normal range (0.287 ng/mL) [24,25]. Data generated during our study demonstrated an association between increased endothelial dysfunction and at least one prior TB episode, and being in a post-TB stage, even in the event of successful treatment. Given recent data that post-TB patients develop pulmonary hypertension [9], which is synonymous with pulmonary vascular angiogenesis, our finding is concerning and suggests that post-TB-related PH may be linked with a pro-angiogenic state. We are not certain what the reason is for this phenomenon in our study. Heart disease, systemic hypertension and HIV status were similar between the populations, suggesting that endothelial dysfunctional status cannot be attributed to these factors. Further investigation is needed. One should keep in mind that the link between TB and angiogenesis is well established, as genes encoding proteins involved in the formation of new blood vessels are known to be expressed during TB infection. However, in our cohort, the majority of the patients had TB 1–5 years ago after being successfully treated for TB and were no longer symptomatic. In our view, it is unlikely that angiogenic pathways should still be elevated, as seen in our patients. Therefore, we postulate that our data could point towards the start of possible pulmonary vascular remodeling.

Angiopoietin-1 is a growth factor which forms part of a group of signaling pathways that perform their function through the tyrosine kinase receptors (Tie1 and Tie2) [26]. One of the primary processes in which angiopoietin-1 is involved in angiogenesis [27]. Angiopoietin-1 also plays a role in the maintenance and stability of the endothelium, functioning as a regulatory molecule by modulating the extent of vascular permeability, inflammation and the degree of angiogenesis [27]. Compared to the literature, the mean angiopoietin-1 in our patients was 6500 times higher than the normal range (0.248 ng/mL) [28]. This supports the inferences drawn from the other markers measured in our population; the post-TB state associates with endothelial dysfunction and lung vascular angiogenesis or vascular remodeling. Again, the concern is that these patients in our study had TB many years ago; therefore, the elevated angiopoietin-1 is troubling.

In terms of the classic markers for cardiac damage and pulmonary hypertension, NT pro-BNP belongs to a family of natriuretic peptides which are produced by the ventricular myocardial cells. It plays a compensatory role in facilitating systemic arterial vasodilatation, natriuresis and diuresis as well as neuromodulation and the actions of the renin-angiotensin-aldosterone [29]. The activity of NT pro-BNP is increased during periods of long-term ventricular overload. It is primarily used as a marker in the diagnosis of heart failure but has a key role in pulmonary hypertension [29]. In our study, NT pro-BNP and cardiac troponin-I were undetectable. This could mean that the patients were not clinically in right heart failure. Therefore, one year after successful TB treatment, these patients may have circulatory signs of some form of vascular remodeling, but they certainly do not have elevated biomarkers for heart disease or cardiac failure. However, one must keep in mind that these markers were measured in the circulatory system, and they may, therefore, not give a direct impression of an altered physiology in the pulmonary vasculature.

There was a positive linear relationship between the number of previous TB episodes and angiopoietin-1, as well as between levels of VEGF and the number of previous TB episodes. The other biomarkers (ADMA, VCAM-1 and TBARS) conversely exhibited weak and negative linear associations with the number of previous TB episodes. As mentioned, both angiopopietin-1 and VEGF play a role in angiogenesis. The data, therefore, suggest that more TB episodes experienced are associated with an increased circulation of vascular remodeling markers. Negative linear correlations were observed between certain biomarkers and the time since the last TB treatment was complete. We observed a negligible negative linear association between the time of the last treatment completion and angiopoietin-1, VEGF and ADMA. This suggests that the levels of these markers are higher in individuals who recently completed their TB treatment and decline as time has elapsed since their last treatment. VCAM-1 and TBARS, on the other hand, displayed a weak yet positive linear relationship with the time since the last TB treatment. Suggesting that their link with this endpoint is negligible. Although we did not pursue statistical correlations between the biomarkers and smoking status or sex, previous research did indicate that these are potentially modifying factors [9]. Regardless, this paper demonstrates the unique role of the number of previous TB episodes and the time since the completion of the last TB treatment in this uniquely elevated risk of endothelial dysfunction and vascular remodeling.

Limitations of this study were that (1) no adequate age-matched healthy controls were included in the parent study, which therefore made comparisons challenging to draw highly accurate inferences. Moreover, (2) it may have been more insightful to have one active TB population that was followed up during and after TB treatment to better establish the full involvement of endothelial pathways. Seeing that most of the endothelial markers were similar between the active TB and post-TB populations, it could create the impression that there was no endothelial dysfunction/angiogenesis. This would be an incorrect assumption. Therefore, it was important for us to evaluate how the concentrations in our study population compare with those of healthy individuals, i.e., in the absence of diseases, as described in previous studies. However, it should be acknowledged that this comparison could be influenced by confounding factors such as genetic backgrounds, sex, age and statistical variation due to sample size. Also, the biomarkers were measured in serum, and although they reflect events in circulation, it does not necessarily mean that there is damage in the pulmonary circulation. Regardless, it is striking to see that a post-tuberculosis state harbors such a unique profile of endothelial dysfunction markers. In light of our findings, it may be important to start following up on patients who have completed their TB treatment. Longitudinal studies can be conducted to better describe the scope of post-TB lung disease and the associated vascular changes. Such an approach could also allow for the earlier identification of patients who are at risk of vascular remodeling and for the start of anti-inflammatory therapy if needed.

## 5. Conclusions

Our findings suggest that having at least one previous TB episode and being in a post-TB stage of the disease, despite successful treatment, is associated with increased endothelial dysfunction/angiogenesis, which may speak to a possible predisposition to pulmonary vascular remodeling and consequently the possible development of pulmonary hypertension, a catastrophic form of post-tuberculosis lung disease. More attention should be given to TB patients in terms of follow-up or longitudinal care to closely monitor endothelial function and to avoid more serious long-term lung complications, such as pulmonary hypertension. Follow-up and long-term care remain some of the challenges in low-income countries such as South Africa due to limited access to healthcare facilities which possess the necessary resources to provide these services. Also, in terms of possible preventive therapy, attention should be given to patients who had multiple previous TB episodes despite having completed TB treatment a long time ago. Future longitudinal studies could better delineate the prevalence and pathophysiology of pulmonary vascular remodeling in post-TB lung disease.

## Figures and Tables

**Table 1:** Descriptive patient data of the study populations, including age (years), sex, smoking history, HIV status, hypertension and known heart disease.

	Post-TB Patients (n = 40)
Age	45.38 ± 13.4
18–29	4 (10.0%)
30–39	10 (25.0%)
40–49	9 (22.5%)
50–59	12 (30.0%)
≥60	5 (12.5%)
Sex	
Female	15 (37.5%)
Male	25 (62.5%)
Smoking status	
Current	21 (52.5%)
Ex-smoker	13 (32.5%)
Never	6 (15.0%)
HIV positive	5 (12.5%)
Hypertension	11 (27.5%)
Known heart disease	6 (15.0%)
TB episodes	
1	25
2	5
3	3
>3	4
Time after TB treatment completion	
1–5 yrs	21
5–10 yrs	9
10–15 yrs	3
>15 yrs	3

**Table 2:** Descriptive summary of the distribution of the various biomarkers.

Parameters	N	Mean	St. Dev.	Min	Max
Angiopoietin-1 (ng/mL)	40	1391.098	733.165	236.600	3226.300
VEGF (ng/mL)	40	562.108	453.787	68.700	2193.900
ADMA (ng/mL)	40	693.638	92.283	547.700	883.100
VCAM-1 (ng/mL)	40	62.275	11.837	25.200	83.500
TBARS (μmol/mL)	40	0.448	0.060	0.310	0.610

**Table 3:** Correlation between the biomarkers and number of previous TB episodes.

	Angiopoietin-1	VEGF	ADMA	VCAM-1	TBARS	Number of Previous TB Episodes
Angiopoietin-1		0.430	−0.092	0.056	−0.014	0.321
VEGF	0.430		−0.142	0.129	0.150	0.386
ADMA	−0.092	−0.142		−0.507	0.120	−0.030
VCAM-1	0.056	0.129	−0.507		0.103	−0.042
TBARS	−0.014	0.150	0.120	0.103		−0.299
Number of previous TB episodes	0.321	0.386	−0.030	−0.042	−0.299	

Computed correlation used the Pearson method with a pairwise deletion.

**Table 4:** Correlation among biomarkers and Time since completion of last TB treatment.

	Angiopoietin-1	VEGF.A	ADMA	VCAM-1	TBARS	Time Since Completion of Last TB Treatment
Angiopoietin.1		0.430	−0.092	0.056	−0.014	−0.162
VEGF.A	0.430		−0.142	0.129	0.150	−0.017
ADMA	−0.092	−0.142		−0.507	0.120	−0.036
VCAM-1	0.056	0.129	−0.507		0.103	0.141
TBARS	−0.014	0.150	0.120	0.103		0.021
Time since completion of last TB treatment	−0.162	−0.017	−0.036	0.141	0.021	

Computed correlation used the Pearson method with a pairwise deletion.

## Data Availability

Patient data is unavailable due to privacy or ethical restrictions, however, anonymized data for the biomarkers can be requested from G.J.M.
